# Social role of the ‘Bow-Wow Patrol’ in urban areas of Japan: a qualitative study

**DOI:** 10.1038/s41598-024-64079-4

**Published:** 2024-06-07

**Authors:** Yoshino Hosokawa, Kaori Ishii, Ai Shibata, Hiroko Yako-Suketomo, Riki Suko, Koichiro Oka

**Affiliations:** 1 Faculty of Health and Sports Sciences, Toyo University, Tokyo, Japan; 2 Faculty of Sport Sciences, Waseda University, Saitama, Japan; 3 Faculty of Health and Sport Sciences, University of Tsukuba, Ibaraki, Japan; 4 Faculty of Physical Education, Japan Women’s College of Physical Education, Tokyo, Japan

**Keywords:** Health care, Medical research

## Abstract

The ‘Bow-Wow Patrol (BWP)’, established to avert local crime by encouraging dog walking, may help build social relationships among individuals through dog walking. However, details of its social influence remain unclear. Thus, this study aimed to elucidate the social roles of BWP in the urban areas of Japan. A total of 18 BWP organisation members from two Tokyo municipalities were recruited using snowball sampling between November 2021 and July 2022. In an interview, participants were asked about their perceptions of changes in their social relationships through the BWP. Qualitative content analysis was conducted after text mining using the KH Coder software. The mean age of the participants was 63.9 years, and 83.3% engaged in BWP 7 days a week. The content analysis revealed six social roles of the BWP: establishment of social networks with schools as the centre, establishment of loose networks inside and outside the BWP organization, enhancement of a sense of trust among neighbours, enhancement of a sense of trust in communities, norms of reciprocity among dog owners, and dog and owner characteristics. This study found that the BWP in urban Japan strengthens social cohesion and expands social networks among dog walkers, encouraging them to continue walking.

## Introduction

Walking provides health benefits for dog owners^1,2^. Some reviews have identified a positive association between the amount (the resultant of the duration times the frequency) of dog walking and owners’ moderate-to-vigorous intensity of physical activity^3–6^. Previous studies in the U.S. and Japan have reported that dog walkers are more likely to meet public health physical activity recommendations^7,8^. The numerous health effects of regular physical activity, such as reduced mortality risk and development of chronic diseases, are well established^9,10^.

As another favourable contribution to dog owners’ health, walking their dogs enhances their social capital, including social interactions, commitment to cooperation, and interpersonal trust^11–18^. Putnam defined social capital as ‘connections among individuals, social networks, and the norms of reciprocity and trustworthiness that arise from them’; it is one of the most widely used^19^. For instance, a study conducted in urban regions of Australia found that dog owners had many opportunities to participate in community events and learn about people living in the suburbs while walking their dogs^16^. Another study conducted in four cities in the U.S. and Australia indicated that dog walkers had more trust in and friendliness toward their neighbours^18^. A previous survey of Japanese populations showed that young to middle-aged dog owners who walked their dogs had significantly more frequent social interactions with neighbours than non-dog owners^13^. Systematic reviews have confirmed that higher individual levels of social capital have several beneficial effects on health outcomes such as all-cause mortality, non-communicable diseases, and mental health^20–23^. However, only a few studies have examined the association between dog walking and owners’ social capital. Thus, further studies are needed to provide evidence promoting dog walking among non-walking dog owners, who account for 36% of all Japanese dog owners^24^.

Dog walking may play an important role in fostering social capital and its elements for owners and their neighbours or community, leading to community-level health benefits. A multi-site international study from the U.S. and Australia revealed that dog walkers in the U.S. had a higher perception of neighbourhood natural surveillance than non-dog walkers. Thus, this suggests that dog walking could increase community perceptions of neighbourhood safety^25^. Moreover, a Canadian study reported that dog walking among older adults was associated with a higher sense of community^26^. Even though social capital is a complex construct, there were quite a few previous studies from Western countries examining the role of dog walking on social capital at the community level or/and its elements. Thus, the detailed roles or mechanisms of dog walking relevant to social capital in neighbourhoods and communities are largely unknown, specifically in Asian contexts characterised by high population densities and cultural contexts that differ from those of Western areas. Considering the positive influence of health and health behaviours on higher community-level social capital—such as smoking cessation behaviour, functional ability, and mental health^27–29^—and the large proportion of the community that owns dogs^30^, an in-depth analysis of how dog walking contributes to building social capital would provide valuable insights. These insights could inform the development of public health recommendations for specific interventions to enhance community social capital through dog walking. Furthermore, applying qualitative research methods to explore the elements and mechanisms of social capital associated with dog walking would be informative in contextualising previous quantitative findings, making them more relevant and specific^31^.

In Japan, a national existing initiative, ‘Bow-Wow Patrol (BWP)’, is a volunteer activity for community safety through dog walking^32^. Each municipality has created unique organisations with dogs and their owners belonging to the BWP are encouraged to watch for children and older adults in the neighbourhood during dog walks. A qualitative exploration of the social role of the existing social initiatives utilising dog walking as an example in their neighbourhoods may help understand the role or some of the mechanisms of dog walking in fostering the social capital among dog owners and communities. Thus, this study aimed to qualitatively clarify the influence of BWP participation among dog owners and the social roles of BWP in the urban areas of Japan by conducting text mining and content analysis using interview data obtained from dog owners participating in BWP.

## Methods

### Study design

This study adopted a quantitative text analysis that integrated quantitative and qualitative data. Quantitative text analysis organises or analyses text-type data using quantitative methods for content analysis^33^. The advantages of using content analysis for this exploratory qualitative study include its suitability for providing a complete overview of a large amount of text data^33–35^. Initially, text mining was used to gain a quantitative and visual overview of the full-text data, followed by a qualitative derivation of categories representing the social role of BWP by intensively reading the original data to gain a deeper understanding of the implications of mechanical data classification.

### Data source and participants

Data were drawn from semi-structured interviews with 18 BWP organisation members in two Tokyo municipalities recruited through purposive snowball sampling from November 2021 to July 2022. Recruitment was terminated when additional data collection no longer yielded new insights or information. Tokyo prefecture was selected as the study area because urban areas tend to have weak social capital owing to the heterogeneity and mobility of the population, which is a significant public health issue that supports activities that contribute to fostering social capital in urban areas^36,37^. Participants were eligible if they had at least one dog and had engaged in the activity at least twice in 12 months or longer. After receiving an explanation of the study’s purpose and agreeing to cooperate with the recruitment of this study, the representatives of the BWP organisation notified their members of the call for participation in the study. Then, with the permission of those interested in participating in this study, the BWP organisation representatives provided their contact details to the author. Subsequently, the author explained the details of this study via phone or face-to-face to prospective participants. The statement distributed to each participant explained the purpose of the study, stated that participation was voluntary, and noted that all transcripts were anonymised. Informed consent was obtained from all the participants included in this study. The participants were offered bookstore gift cards (1000 JPY, equivalent to approximately 8 USD) as compensation for their time.

### Measures

Data on sociodemographic attributes and dog- and BWP-related variables were obtained using a questionnaire. Sociodemographic characteristics included sex, age, living with family or other cohabitants, type of housing, and administrative units of the participants. The municipality’s administrative unit was the smallest organisational unit in Japan (city, suburb, town, or village). Dog characteristics included dog sex, dog age, history of dog ownership (years), dog weight (kg), dog-keeping places, participation in dog training classes, and the number of dogs owned. BWP-related variables included years of BWP experience (years), frequency of dog walking per day (number), total time spent walking per day (minutes), and number of dogs walking per week (number). Given that the BWP is performed during regular dog walking, the time of regular dog walking was ascertained as the time of BWP.

Semi-structured interviews were conducted to identify the perceived social role of BWP activities. The interview guide was designed with the following questions to accomplish the primary objectives: the reasons for participating in the BWP; what they paid attention to during the BWP; the physical, mental, and social changes they had experienced through the BWP; how the BWP helped the community; and the issues of the BWP. A reference group of public health stakeholders checked the interview guide and study protocol (four members with backgrounds in health promotion, behavioural health science, and health education).

### Statistical analysis

The interviews were audio recorded and transcribed verbatim for analysis. This study used KH Coder version 3, text-mining software, to analyse the interview data^38^. KH Coder has been used to analyse text data from various genres, including interviews, social networking services, mass media, and social surveys^33^. The authors imported the qualitative text data into a computer and then used morphological analysis to break the data into words. This process decomposed the language into smaller segments, classifying morphemes—minor meaningful language units—into parts of speech.

Second, frequently occurring words and keywords were extracted and their frequency and simultaneous occurrence relationships were analysed. The co-occurrence network diagram shows the frequency of the words and the co-occurrence of two or more words. The strength of the co-occurrence relationship was measured using the Jaccard coefficient, which expresses the relationship between words as a number between 0 and 1, with a coefficient closer to 1 indicating a stronger co-occurrence relationship^39^. The chart suggests stronger co-occurrence relationships using thicker solid lines and depicts words with higher occurrences in larger circles. Morphological and co-occurrence analyses were conducted using KH Coder version 3 (Kyoto, Japan). The analysis was initially performed in Japanese, and the results were translated into English when preparing the research paper. The first author translated the Japanese text, which five co-authors then reviewed. A native English speaker has proofread this manuscript.

A summary content analysis was conducted manually to categorise the groups in the co-occurrence network into categories^40^. First, each group’s textual data containing words was carefully read based on identification using a co-occurrence network. From these text data, meaningful sentences regarding the social role of BWP were extracted and organised as codes using standard terms. Similar codes were then gathered into the overarching subcategories. Finally, similar subcategories were summarised and named. Four authors from diverse backgrounds analysed and coded the data to ensure confirmability. Subsequently, this process was carefully reviewed and refined until all authors reached a consensus. To enhance the credibility of the results, three participants performed member checks to ensure no discrepancies in the interpretation of the results.

### Ethical approval

The study was conducted in accordance with the Declaration of Helsinki and approved by the Research Ethics Committee of Waseda University, Japan (2021-333).

### Informed consent

All the participants signed an informed consent form before participating in the interviews.

## Results

### Characteristics of the study participants

Table 1 shows the characteristics of the study participants. A total of 72% of participants were female. The mean age was 63.9 (SD 6.4) years. They all lived with family or other cohabitants; 72.2% lived in owner-occupied houses, and 61.1% lived in the suburbs. More than two-thirds of their dogs were female, the mean age of the dogs was 7.7 (SD 4.5) years, the history of dog ownership was 7.2 (SD 4.7) years, and the dog weight was 11.3 (SD 8.7) kg. All dogs were kept indoors, 66.7% attended training classes, and participants had an average of 1.2 (SD 0.5) dogs. Participants averaged 12.7 (SD 5.3) years of BWP experience. On average, participants walked with their dog 1.8 (SD 0.9) times per day, 71.1 (SD 42.1) min of total dog walking per day, and 6.2 (SD 2.0) times dog walking per week.

**Table 1:** Characteristics of the study participants (N = 18).

Sociodemographic measures		
Sex, n (%)		
Male	5	(27.8)
Female	13	(72.2)
Age, years, mean (SD)	63.9	(6.4)
Living with family or other cohabitants, n (%)		
Yes	18	(100.0)
No	0	(0.0)
Type of housing, n (%)		
Owner-occupied house	13	(72.2)
Owner-occupied apartment	3	(16.7)
Rented house	2	(11.1)
Rented apartment	0	(0.0)
Administrative unit, n (%)		
City	7	(38.9)
Suburb	11	(61.1)
Dog characteristics measures		
Dog sex, n (%)		
Male	6	(33.3)
Female	12	(66.7)
Dog age, years, mean (SD)	7.7	(4.5)
History of dog ownership, years, mean (SD)	7.2	(4.7)
Dog weight, kg, mean (SD)	11.3	(8.7)
Dog keeping places, n (%)		
Indoors	18	(100.0)
Outdoors	0	(0.0)
Participation in dog training classes, n (%)		
Yes	12	(66.7)
No	6	(33.3)
The number of dogs owned, mean (SD)	1.2	(0.5)
The BWP-related measures		
Years of BWP experience, years, mean (SD)	12.7	(5.3)
Frequency of dog walking per day, number, mean (SD)	1.8	(0.9)
Total time of dog walking per day, minutes, mean (SD)	71.1	(42.1)
Number of dog walking per week, number, mean (SD)	6.2	(2.0)

Sample: eighteen BWP organization members in two Tokyo municipalities.

*BWP* Bow-Wow Patrol, *SD* standard deviation.

### Social roles of the BWP

Table 2 presents a list of the frequently used words. From the morphological analysis, 1508 words were extracted from the textual data. The most commonly mentioned word was ‘dog’, followed by ‘people’, ‘think’, ‘say’, and ‘BWP’ in that order.

**Table 2:** Word occurrence frequency in text data.

Rank	Word	Frequency
1	Dog	377
2	People	309
3	Think	292
4	Say	192
5	BWP	135
6	Keep	127
7	Enter	118
8	Children	115
9	Dog walking	99
	My baby dog	99

**Figure 1:**
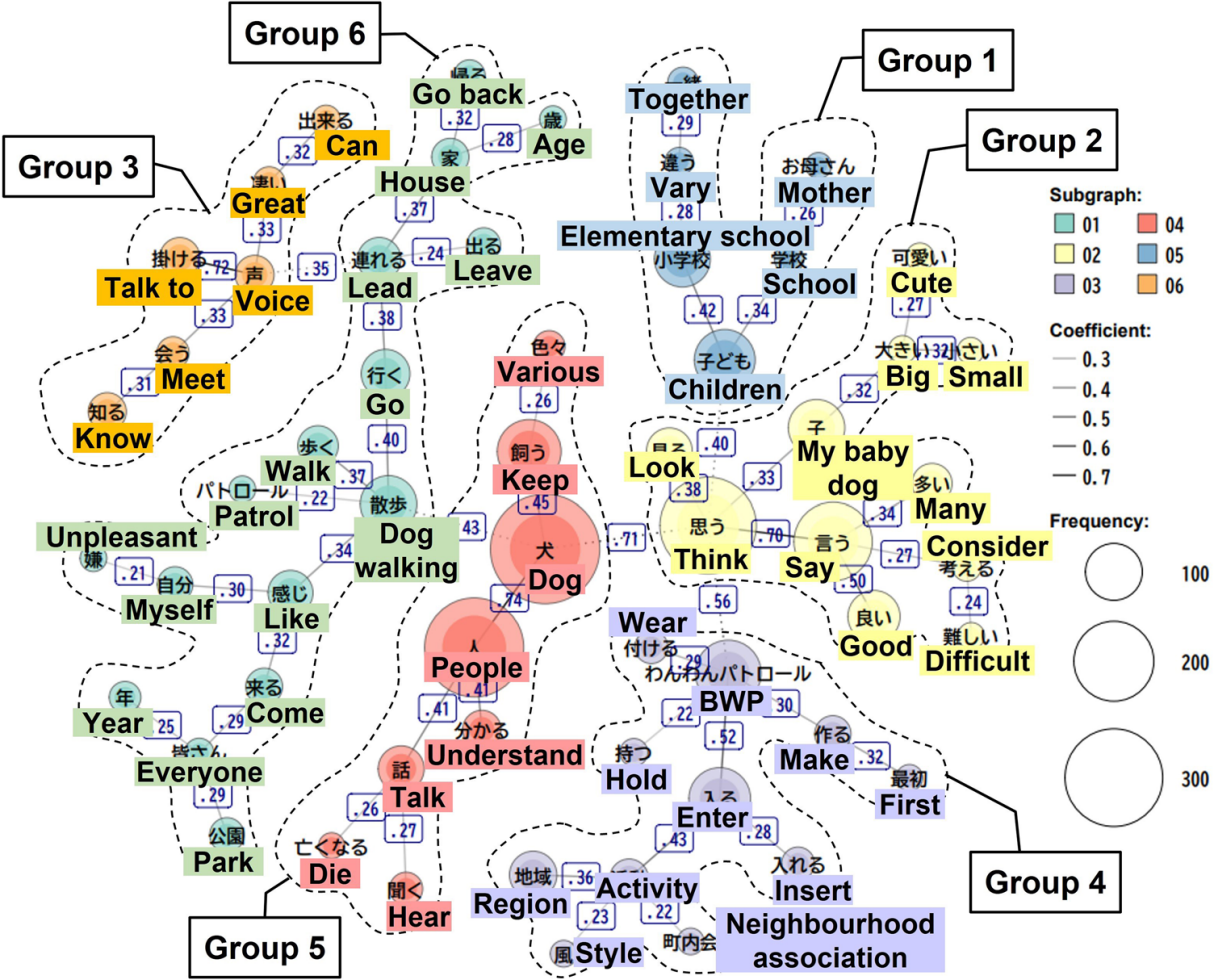
Co-occurrence network of high-frequency words in text data (part of the figure was created with KH Coder version 3).

The results of the co-occurrence network analysis automatically classified the words into six groups of 6–16 words (Fig. 1). The words that showed characteristic co-occurrence in each group were as follows:

Group 1 had a high coefficient connecting ‘elementary school’ and ‘children’ (Jaccard coefficient = 0.42) and co-occurred with words such as ‘school’ (0.34) and ‘mother’ (0.26). Group 2 showed a strong co-occurrence of ‘think’ and ‘say’ (0.70) and derivation to the words ‘look’ (0.38), ‘my baby dog’ (0.33), ‘big’ (0.32), and ‘small’ (0.32). Group 3 confirmed the high co-occurrence of ‘voice’ and ‘talk to’ (0.72) and co-occurred with words such as ‘meet’ (0.33) and ‘know’ (0.31). Group 4 spreads out from ‘BWP’ to ‘enter’ (0.52), ‘activity’ (0.43), and ‘region’ (0.36). Group 5 had a high coefficient connecting ‘dog’ and ‘people’ (0.74) or ‘keep’ (0.45) and co-occurred with words such as ‘understand’ (0.41) and ‘talk’ (0.41). Group 6 confirmed the high co-occurrence of ‘dog walking’ with ‘go’ (0.40), ‘walk’ (0.37), or ‘lead’ (0.38).

The content analysis results, conducted to name the groups of co-occurrence networks as categories, are presented in Table 3. The following section provides a description and representative quotations for each category. A total of 32 codes identified as having social roles in the BWP were grouped into 15 subcategories. Similar subcategories were further summarised, and the following six categories were identified.

**Table 3:** Social role of the "Bow-Wow Patrol" in urban areas of Japan.

Categories	Subcategories	Codes	Number of meaning unit	Groups
Establishment of social networks with schools as the centre	Relationships with neighbourhood children	Watch over the safety of children in the neighbourhood	17	Group 1
		BWP provides opportunities to interact with neighbourhood children	27	
	BWP activities with neighbourhood schools as the centre	Collaboration with schools will clarify the purpose of BWP activities	21	
		BWP will create intergenerational communication with the school as the centre	11	
Establishment of loose networks inside and outside the BWP organisation	Low-burden activities for the BWP members	The loose network facilitates the continuation of activities	13	Group 2
		Watch over the neighbourhood during routine dog walks	19	
	Building a society where dogs and people coexist harmoniously	Differences in engagement depend on the type and age of the dogs	26	
		Care for neighbours and their dogs to live in harmony	12	
Enhancement of a sense of trust among neighbours	Accelerating communication among neighbours	They were walking with dogs and being approached by neighbours	18	Group 3
		Walking with a dog makes it easier to talk to neighbours	17	
	Creating interaction with neighbours through dogs	People who have dogs get to know each other	12	
		Get to know neighbours who are not dog owners	7	
	Sharing information about the BWP	They knew about BWP activities before they got the dog	7	
		A desire to make others aware of BWP's activities	9	
	Developing proactive community activities	Organize dog-related events with BWP members	6	
		They are developing BWP activities proactively	4	
Enhancement of a sense of trust in communities	Fostering a sense of community	There is a sense of watching over the safety of our community	22	Group 4
		BWP is an excellent opportunity to get people interested in the community	10	
	Characteristics of dog-friendly communities	Obtain support for BWP activities from public agencies	7	
		Collaborate with the chairperson of the neighbourhood association and school principal	12	
		Dog owners have their favourite parks	21	
	Strengthening social cohesion among dog owners	They wear matching merchandise to walk their dogs	26	
		The relationship between dog owners becomes more intimate	12	
Norms of reciprocity among dog owners	Dog owners are willing to help each other	Dog owners notice changes in each other	10	Group 5
		They are concerned about the ageing of dogs and their owners	20	
		They worry about their physical and mental decline after losing their dogs	11	
	Have a strong sense of responsibility for dog ownership	Responsible for dog ownership and walking	7	
		Recognize that their dogs are a problem for others	23	
Dog and owner characteristics	Voluntary behaviour that results from attachment to the dogs	They do not require BWP members to patrol neighbourhoods	15	Group 6
		BWP members do not feel burdened by dog care	16	
	Activity style according to dog and owner characteristics	They see specific people in the parks they routinely go to with their dogs	16	
		Activity levels of dog owners are affected by their dog's characteristics	26	

### Establishment of social networks with schools as the centre

BWP members walked with their dogs around the school at times that coincided with neighbourhood children’s arrival and departure from school and networked with children and their parents. This category comprised two subcategories: relationships with neighbourhood children and BWP activities with neighbourhood schools as the centre. These subcategories were summarised and named ‘establishment of social networks with schools as the centre’.

‘When I walk with my dog around the neighbourhood elementary school, the children greet my dog every morning’. (Participant 1)

‘We even had elementary school children create posters introducing the BWP and bandanas for dogs as a part of their classes. That way, the parents of the children would know about the BWP’. (Participant 2)

### Establishment of loose networks inside and outside the BWP organisation

Establishing loose networks inside and outside the BWP organisation is essential for maintaining BWP activities. This category has two subcategories: low-burden activities for the BWP members and building a society in which dogs and people coexist harmoniously. BWP members were aware of neighbours’ discomfort with dogs and made various efforts to help them coexist with their dogs. The two subcategories were named ‘establishment of loose networks inside and outside the BWP organisation’.

‘If I did not have a dog, I would not take walks. Sometimes I notice small changes because I have to walk with my dog daily in the neighbourhood’. (Participant 3)

‘There are many different types of dogs in BWP, from small to large, and all owners need to be aware that even if their dogs are small, they may be a nuisance to neighbours’. (Participant 4)

### Enhancement of a sense of trust among neighbours

This category had four subcategories: accelerating communication among neighbours, creating interaction with neighbours through dogs, sharing information about the BWP, and developing proactive community activities. The BWP members felt that walking with their dogs facilitated two-way communication with their neighbours. It was confirmed that increased communication enhances community residents’ trust, and that BWP activities are becoming more active. These subcategories were named ‘enhancement of a sense of trust among neighbours’.

‘When I walk with my dog, dog owners and non-dog owners talk to me’. (Participant 5)

‘If I did not have my dog with me, I would have been suspicious when I approached the neighbourhood children and would not have had the opportunity to communicate with them’. (Participant 6)

‘We used to gather at a park that BWP members frequent and hold a dog training class inviting an instructor’. (Participant 7)

### Enhancement of a sense of trust in communities

This category includes three subcategories: fostering a sense of community, characteristics of dog-friendly communities, and strengthening social cohesion among dog owners. BWP members enhanced their social cohesion by wearing matching merchandise featuring dog patrols. Dog owners developed greater trust in their communities, through BWP activities. They also had a strong sense of community and were committed to creating a comfortable environment for dog ownership. The three subcategories were summarised as ‘enhancement of a sense of trust in communities’.

‘Dog owners in the neighbourhood who used to be only acquaintances have become very close since entering BWP’. (Participant 8)

‘I think it is meaningful for men to participate in activities such as the BWP and stay connected to the community before they retire from work’. (Participant 9)

‘The administration and BWP members cooperate to maintain the park’s environment, making it easier for dogs to play’. (Participant 10)

### Norms of reciprocity among dog owners

This category was named after two subcategories: dog owners who were willing to help each other, and had a strong sense of responsibility for dog ownership. BWP members became friends with their neighbours by walking with their dogs and building relationships of mutual help between dog owners and neighbours who did not own dogs. These subcategories were named ‘norms of reciprocity among dog owners’.

‘Sometimes, I comfort the owner of someone’s dog whom I always met at BWP when the dog has passed away’. (Participant 11)

‘When my husband was hospitalised and away from home, a neighbour was very helpful in caring for our dog’. (Participant 12)

### Dog and owner characteristics

The category included two subcategories: voluntary behaviour resulting from attachment to dogs, and activity style according to dog and owner characteristics. The frequency of walking with dogs on patrols and how they interacted with other dog owners was related to dog type and the owner’s attachment to the dogs. The two subcategories were summarised as ‘dog and owner characteristics’.

‘There is no need to feel obligated to patrol your neighbourhood just because you have joined the BWP; enjoy walking with your dog and care about the neighbourhood a little’. (Participant 13)

‘My dog is elderly and cannot walk a lot, but just standing in front of my house with my dog and saying hello to the children is a patrol’. (Participant 14)

## Discussion and conclusion

This study aimed to determine the influence of participation in the BWP on dog walking behaviour and social relationships between dog owners and their neighbours in the urban areas of Japan. The participants in this study reported walking their dogs approximately twice a day for 1.2 h daily. Similarly, a previous study in Japan showed that 191 older Japanese dog walkers walked for an average of 56 min per day^41^. Earlier studies in the U.S. and Australia also reported that dog walkers (n = 623) walked with their dogs for 93–109 min/week^25^. The high dog-walking time of the owners in this study could be due to the study sample size, regardless of the study countries conducted. In addition, participating in BWP activities instead of simply walking a dog may affect the amount of dog walking. The social interactions gained through BWP activities contributed to the maintenance and promotion of walking with dogs among the participants in this study^42^. Furthermore, the variation in dog walking time between Japan and Western countries is likely because the neighbourhood-built environment has some effect^43–45^, although methodological differences exist. The participants in this study lived in walkable, high-density urban areas, which may have supported walking with their dogs^46^.

Content analysis revealed six categories of social roles in the BWP, indicating that dogs act as catalysts for social interactions. The categories obtained in this study can be explained using components of social capital^19^. The categories such as ‘Establishment of social networks with schools as the centre’ and ‘Establishment of loose networks inside and outside the BWP organisation’ implying connections among individuals and social networks were obtained. They highlighted that BWP members walked with their dogs around the school and built a network with neighbourhood children and their parents. The present findings support earlier Australian and U.S. studies that concluded that casual interactions facilitated by dog walking enhance social networks^17,18,42^. One unique aspect of dog walking is that previous research has reported that walking with dogs along the same path at approximately the same time each day may promote friendship formation^17^. BWP members widely recognise neighbourhood children because of their habit of walking dogs when they arrive and depart from school. Ongoing intergenerational interactions between older citizens and children can strengthen neighbourhood trust^47^. However, it is undeniable that negative perceptions of dog behaviour often extend to owners^48^. BWP members were conscious of establishing a loose connection, considering the differences in how they interacted with dogs of different types and ages and neighbours uncomfortable with dogs.

Moreover, neighbours and local governments positively recognised BWP members for wearing matching bandanas and bags with dog patrol motifs. The unique activity style of BWP might have fostered a sense of community among owners and supported the ‘Enhancement of a sense of trust among neighbours and communities’ and ‘Norms of reciprocity among dog owners’. The results of this study contribute to the existing literature by suggesting a positive relationship between dog walking and higher levels of social capital, including trust and reciprocity^17,18^.

Fostering a sense of community among owners through BWP encouraged them to walk daily with their dogs^26^. Other factors related to continued dog walking behaviour include owners’ sense of obligation, self-efficacy in dog walking, and attachment to their dogs^7,45,49,50^. The category ‘Dog and owner characteristics’ showed that the BWP members did not feel burdened by dog care and emphasised voluntary activities that result from attachment to their dogs. Furthermore, BWP members enjoyed seeing and conversing with specific individuals in parks they routinely visited with their dogs. Small-scale parks provide opportunities for dogs to play and interact with other dogs and for human visitors to interact socially with other dog owners^12^. A cross-sectional study conducted in the United States found that the perception of social support from friends was strongly related to self-efficacy during dog walking^51^. Cooperative relationships among BWP members may reinforce self-efficacy in dog walking. Some studies have indicated that not all dog owners walk their dogs with attachment^13,41,50,52^. Dog characteristics have also been found to influence dog walking, with older dogs being less active than younger dogs, and larger dogs being more active than smaller dogs^53^. Further research is required to confirm how dog and owner characteristics influence the development of social relationships through dog walking.

This study has some limitations. In particular, since the focus is on BWP activities in two municipalities in Tokyo, careful attention should be paid to applying the results of this study to other municipalities. The design of the surrounding built environment has been reported to facilitate or hinder the expected health benefits of walking for dogs^54–56^. Therefore, it is necessary to examine whether the findings of this study can be applied to other geographical contexts in the urban areas of Japan. In addition, owing to the small sample size, differences in the characteristics of the dogs and their owners remain unclear. Those who participated in the interviews may have held more favourable views of social capital and dog ownership. The strength of this study is that specific interventions to build social capital through dog walking were suggested in the less-studied geographical context of Asia. Future research should develop a quantitative study that expands the target areas to clarify the interrelationships among dog walking, owners’ physical activity, and social capital.

This qualitative study revealed that the social role of BWP in urban areas of Japan might strengthen dog walkers’ social cohesion and further promote their social networks through dog walking. Owing to the influence of participating in BWP among dog owners, it was further confirmed that social connections through BWP may encourage owners to continue walking. Constructing a behavioural health intervention through dog walking may foster social capital in the community surrounding dog walkers.

## Data Availability

The datasets used and/or analysed during the current study are available from the corresponding author upon reasonable request.
